# Genetic underpinnings of the psoriatic spectrum

**DOI:** 10.1515/medgen-2023-2005

**Published:** 2023-04-05

**Authors:** Ulrike Hüffmeier, Janine Klima, Mohammad Deen Hayatu

**Affiliations:** Universitätsklinikum Erlangen, Friedrich-Alexander-Universität Erlangen-Nürnberg Institute of Human Genetics Schwabachanlage 10 91054 Erlangen Deutschland; Universitätsklinikum Erlangen, Friedrich-Alexander-Universität Erlangen-Nürnberg Institute of Human Genetics Schwabachanlage 10 91054 Erlangen Germany; Universitätsklinikum Erlangen, Friedrich-Alexander-Universität Erlangen-Nürnberg Institute of Human Genetics Schwabachanlage 10 91054 Erlangen Germany

**Keywords:** Psoriasis vulgaris, psoriatic arthritis, pustular psoriasis, *HLA*, *IL36RN*

## Abstract

The psoriatic field includes both rare and common subtypes. Common complex forms include psoriasis vulgaris and psoriatic arthritis. In these subtypes, certain *HLA* alleles remain the most relevant genetic factors, although genome-wide association studies lead to the detection of more than 80 susceptibility loci. They mainly affect innate and adaptive immunity and explain over 28 % of the heritability. Pustular psoriasis comprises a group of rarer subtypes. Using exome sequencing, several disease genes were identified for mainly generalized pustular psoriasis, and an oligogenic inheritance is likely. Treatment studies based on the affected IL–36 pathway indicate a high response rate in this subtype further supporting the pathophysiological relevance of the affected gene products.

## Epidemiology and clinical features of psoriasis

Psoriasis is a chronic, inflammatory skin disease. As a multifactorial disease, psoriasis is caused by both, genetic and environmental risk factors [1, 2]. Accounting for about 90 % of all cases, **psoriasis vulgaris (PsV)** is the most common type of psoriasis. It has an estimated prevalence of 2–4 % in Western countries, whereas there is some variation due to geography, ethnicity and other environmental and genetic factors. In Europe, the highest prevalence has been reported for Norway with 4.8 and 8.5 %. For the United Kingdom, on the other hand, PsV prevalence was reported to be 1.3 to 2.2 %, respectively [3]. It is characterized by well-delineated, erythematous oval plaques with adherent white to silvery scales. The scales are a consequence of hyperproliferation of epidermal cells and parakeratosis (abnormal maturation of keratinocytes and persistence of nucleated cells in the uppermost epidermal layer). In affected skin, the mitotic activity of basal keratinocytes is increased, resulting in acanthosis (epidermal thickening). In addition, dermal inflammatory infiltrate affects overall thickness of the lesions. While the inflammatory infiltrate in the dermis mainly consists of dendritic cells, macrophages and T cells, in the epidermis, it is composed of neutrophils and to a smaller extent T cells. The red coloration, observed in psoriatic lesions, is caused by increased visibility of tortuous capillaries [1].

The most common comorbidity of PsV is **psoriatic arthritis (PsA)**. As a spondyloarthropathy, it is characterized by systemic musculoskeletal inflammation, typically manifesting at peripheral joints, the spine, and/or entheses [4]. Approximately 30 % of patients suffering from PsV additionally develop PsA, while, 90 % of the majority of PsA patients (90 %) have concomitant PsV [5].

To a large extent, psoriasis susceptibility can be attributed to a person’s genetic makeup and several studies have pointed to familial aggregation of psoriasis worldwide [6, 7]. Twin studies indicated that the risk of developing psoriasis was two to three times higher in monozygotic than in dizygotic twins, while the concordance rate for psoriasis was 33 % in monozygotic and 17 % in dizygotic twins [6]. Two more recent studies estimated a comparable **heritability** h^2^_SNP_ for PsA and PsV: Soomro and colleagues reported a heritability of 0.61 and 0.63, respectively [8], while Li et al. calculated a heritability of 0.41 and 0.37, respectively [9]. These values, however, depend on the method of heritability estimation, and a considerable proportion of heritability was contributed by SNPs within the major histocompatibility complex I (MHC I) [9]. No such figures were reported for pustular psoriasis.

Considerably rarer are clinical phenotypes of psoriasis with neutrophilic skin inflammation presenting as macroscopic, aseptic pustules, known as **pustular psoriasis** [10]. These subtypes are heterogeneous and can sometimes occur concomitantly in single individuals. Pustular psoriasis and PsV can also manifest together, and discussions are ongoing whether pustular psoriasis should be considered as a subtype of PsV or a separate entity.

**Generalized pustular psoriasis (GPP)** can present as a persistent, highly inflammatory subtype that can be fatal [11, 12]. GPP may also appear as multisystemic, episodic disease characterized by widespread skin erythema with sterile pustules, systemic symptoms and extracutaneous manifestations [13, 14]. A typical histological feature of GPP is an epidermal infiltration of neutrophils and mononuclear cells, more pronounced than in PsV; it manifests as sterile pustules [14]. Depending on course of disease and clinical presentation, different subtypes of GPP were defined: von Zumbusch type, GPP in pregnancy or impetigo herpetiformis, annular, and GPP with PsV [13]. Because GPP is such a rare condition, only a handful of epidemiologic studies have been conducted. It is estimated that the disease affects 1.76 people per million in Western Europe, but for Japan and South Korea, a prevalence of ~7 cases per million was reported, pointing to a potentially higher prevalence in East Asian populations [15–18].

**Acrodermatitis continua of Hallopeau (ACH)** was first described by Francois Henri Hallopeau in 1890 as a painful acral pustular condition characterized by sterile pustules involving distal fingers and to a lesser extent toes. ACH is rare and often debilitating; it can manifest after a single-digit injury or infection [19]. In the early stages of hyperaemia, pustules occur at the tips of one or two fingers and progress proximally; if the nail bed and nail matrix are affected, this can lead to anonychia, onychodystrophy, and the destruction of the nail plate [19]. While some consider it a distinct entity, many consider it a subtype of pustular psoriasis, as there are cases of ACH progressing to GPP [20]. To the best of our knowledge, prevalence figures for ACH just like the following pustular skin disease do not exist.

**Acute generalised exanthematous pustulosis (AGEP)** is a pustular skin eruption caused by adverse reaction to certain drugs such as antibiotics and non-steroidal anti-inflammatory drugs [21]. Strictly, it does not belong to the psoriatic spectrum, but is here considered as relevant, as it resembles GPP genetically and clinically. Despite the similarity of AGEP and GPP in their clinical presentation, slight pathologic distinctions, such as drug induction and a more acute course of fever and pustulosis with rapid spontaneous recovery, may be used to distinguish AGEP from GPP [21]. This reference describes a scoring system recently modified by Paulmann and Mockenhaupt [22] and used by experts in the field to diagnose AGEP.

**Palmoplantar pustulosis (or palmoplantar pustular psoriasis, PPP)** is another rare subtype of psoriasis. The prevalence depends on the ethnicity and ranges between 0.05 % (Sweden) and 0.12 % (Japan), whereas the prevalence in Germany is estimated to be 0.091 % [23]. It is characterized by hyperkeratosis and clusters of sterile, neutrophil-filled pustules on palms and soles. PPP occurs more often in women than in men with a proportion ranging from 58 to 94 %. Smoking is a known important contributing environmental factor: 42 to 100 % of patients are current or past smokers. The mean age of onset is 48 years. Between 14 to 61 % of PPP patients have concomitant PsV. The prevalence of PsA among PPP patients ranges from 8.6 to 28 % [23, 24].

## Susceptibility factors for PsV

Genome-wide association studies (GWASs) have been rather successful in the psoriatic field and lead to the identification of **more than 80 susceptibility loci** up to date, as systematically summarized recently [25]. In sum, they explain more than 28 % of the estimated heritability [26]. However, typical limitations of GWAS also apply in psoriasis, e. g. credible SNPs are not necessarily causal, intergenic findings are difficult to interpret, and the putative disease-relevant gene is often only inferred [27]. For most loci, it is unknown how they contribute to disease pathogenesis. Therefore, we will not elaborate on all loci, but rather focus on well-established and better understood genetic risk factors/pathways.

Only two risk loci discovered in the pre-GWAS era were later confirmed by GWAS: the psoriasis susceptibility loci PSORS1 and PSORS2. PSORS1 harbours the main susceptibility factor for PsV. It is widely agreed that HLA-C*06:02 (HLA: human leukocyte antigen) is the susceptibility allele within PSORS1; it represents a haplotype within the *HLA-C* gene at MHC I [28]. The role of this allele in psoriasis pathogenesis is still not fully understood. Nevertheless, its importance is underlined by another susceptibility factor, namely ***ERAP1***. ERAP1 is a protease playing an important role in processing of MHC I peptides. *ERAP1* variants showed an epistatic effect in individuals also carrying the *HLA-C* risk allele [29].

Fine mapping of PSORS2 lead to the identification of susceptibility variants in ***CARD14*** [30]. The caspase recruitment domain member 14 acts as a scaffolding protein that can activate pathways of inflammatory transcription factor NF-κB and p38/JNK MAP kinase signalling [30]. Rare *gain-of-function* variants were identified within the PSORS2 locus in affected members of a Taiwanese family (c.349+5G>A) and in a family with European ancestry (c.349G>A/p.(Gly117Ser)); both familial mutations affected splicing and led to a 22-amino-acid insertion *in vitro* and *in vivo* [30]. Other variants were associated with an increased NF-kB activation and overexpression of psoriasis-associated genes in keratinocytes compared to wild type [31].

The identification of the type I cytokine receptor gene ***IL23R*** as genetic risk factor for psoriasis initially was a secondary finding: Variants at ***IL12B***, a gene encoding a subunit shared by the cytokines IL–12 and IL–23, were found to be significantly associated with psoriasis in a GWAS. Upon analysing sequences of other chains of the cytokines and their receptors, association of two coding variants in *IL23R* were detected [32]. In a later GWAS comprising more than 10,000 individuals, association of *IL23R* variants with psoriasis could be confirmed [33]. IL–23 inhibitors represent effective therapeutics in PsV. The prevailing hypothesis is, that IL–23 facilitates 1) *in situ* proliferation and maintenance of skin resident memory T_H_17 cells (T_RM_17) and 2) promotion of T_H_17 cells. On the one hand, those IL–17 producing cells play a role in skin defence, on the other hand, they also contribute to autoimmune diseases. Anti-IL–23 therapy is assumed to cause a depletion of T_RM_17 cells from lesional skin and thereby improve or even resolve symptoms [34].

Two coding variants in ***TRAF3IP2*** (encoding the TRAF3 interacting protein 2, also known as transcription factor NF-κB activator 1 (Act1)), have been reproducibly found to be associated with PsV. As a signalling adaptor, Act1 plays a role in the regulation of adaptive immunity. For one thing, it is a negative regulator of CD40-BAFF-mediated B cell functions, leading to an inhibitory effect on humoral immune responses. Apart from that, Act1 is essential in IL–17 signalling [35, 36]. Neutrophil extracellular traps (NETs) were shown to stimulate the induction of IL–17 producing T_H_17 cells. The coding variant rs33980500 (*TRAF3IP2* c.28C>T/p.(Asp10Asn)) causes a hyperactive T_H_17 response and thus increased levels of IL–17 [37]. IL–17 mediates the interaction between Act1 and UL–17R, leading to the recruitment of the tumor necrosis factor associated factors (TRAF) TRAF3 and TRAF6. The resulting signalling complex subsequently activates MAPK and NF-κB pathways. NF-κB mediates the transcription of various pro-inflammatory cytokines, rendering it a master regulator of innate immunity [35, 36]. *TRAF3IP2* represents one of the few susceptibility loci for which the disease-contributing allele and its pathophysiological role were elucidated.

Many genes mapping to psoriasis loci have **immune-related functions** such as lymphocyte differentiation/regulation, response to virus/bacteria, type I interferon, and regulation of the I-κB kinase/NF-κB cascade. Genes at more than 10 loci function in the NF-κB cascade, underlining the importance of this pathway in psoriasis susceptibility. Focussing on immune cells, association signals are especially enriched in enhancers, which are active in CD4^+^ T helper (T_H_0, T_H_1 and T_H_17) and CD8^+^ cytotoxic T cells [26]. Most susceptibility loci identified so far reflect the role of innate and adaptive immunity in psoriasis, but not its nature as a skin disease.

## Differing susceptibility factors for PsA

Many of the known susceptibility loci are shared between PsV and PsA, probably due to the presence of psoriasis in both traits [38]. For this reason, we will focus here on PsA-specific genetic risk factors and pathways.

Although the variants described in the following are located within susceptibility loci also for PsV, the alleles themselves are specific for PsA. Within the *HLA* locus, there are some alleles conferring risk specifically for PsA, among them HLA-B*27 [39]. This is also the major genetic variant associated with ankylosing spondylitis, another inflammatory, arthritic disease [2]. Also, within the ***IL23R*** locus, **distinct variants** for PsA or PsV, respectively, were identified [38].

Of the 16 proposed PsA-specific risk loci, only four loci reached genome-wide significance and are discussed here [40]. In a GWAS comprising more than 3,000 PsA patients, a significant association between PsA and the nonsynonymous variant rs2476601 in ***PTPN22*** has been found [41]. This variant has also been reported in other autoimmune diseases including rheumatoid arthritis. The gene encodes the lymphoid protein tyrosine phosphatase (Lyp) and is expressed in hematopoietic cells and immune cells. The missense substitution affects a domain for protein-protein interaction with Csk (C-terminal Src kinase). The resulting complex regulates both B cell and T cell receptors. It is assumed that Lyp activity is enhanced by the risk allele, leading to an inhibition of T cell signalling [42].

Another susceptibility variant maps to an intergenic region between the two genes* CSF2* and *P4HA2* on chromosome 5q31. Functional annotation and gene expression data from CD8^+^ and CD4^+^ T cells was used to identify promising candidate genes within the susceptibility locus, resulting in the identification of rs11955347 in ***SLC22A5***. The variant’s risk allele is associated with decreased *SLC22A5* expression [38]. The gene encodes the carnitine transporter OCTN2 that plays a role in the β-oxidation pathway. It has been shown that OCTN2 levels are increased by cytokine-dependent inflammation [43]. Nevertheless, the PsA-specific association could not be confirmed by a larger GWAS comparing PsA patients with cutaneous-only psoriasis (PsC) patients, instead of comparing them to healthy individuals [44].

In a study examining CNVs associated with PsA, a highly significant association of an intergenic deletion of ~26 kb between ***ADAMTS9*** and ***MAGI1*** was detected. SNPs within the two genes did not show any significant linkage disequilibrium with the deletion, leading to the assumption that the deletion itself is causal. The *ADAM metallopeptidase with thrombospondin type 1 motif 9* (*ADAMTS9*) gene encodes an aggrecanase, an enzyme facilitating the degradation of aggrecan, a cartilage-specific proteoglycan. Cartilage degradation in inflammatory joint disease like PsA was found to be associated with aggrecanase activity, rendering it a promising candidate for further research. Although the membrane-associated guanylate kinase, WW and PDZ domain containing 1 (MAG1) is a molecule previously not implied in immune diseases, it was shown to interact with a signalling protein involved in the negative regulation of regulatory T cells [45].

In a recent GWAS, ***B3GNT2***, a gene involved in the glycosaminoglycan (GAG) metabolism, was found to be associated with PsA. In a subsequent genome-wide pathway analysis, a significant and specific association of the GAG metabolism with PsA was identified. This was confirmed by differential expression analysis of this pathway’s genes in blood of PsA patients compared to controls. GAGs are linear oligosaccharides and play a crucial role in glycosylation of proteins. The combination of the resulting proteoglycans and collagen is a major component of cartilage, a PsA-relevant tissue. It is hypothesized, that variants affecting the GAG pathway could reduce biosynthesis of GAGs, thereby leading to diminished availability for aggrecan and cartilage formation in PsA [40]. Another genome-wide pathway analysis on PsA did not confirm the findings affecting the GAG pathway. Instead, they found an enrichment of PsA-specific variants in genes involved in the NFκB cascade and Wnt signalling as compared to PsC [8].

## Genetic architecture of GPP, ACH and AGEP

While linkage studies and mainly whole exome sequencing have identified deleterious variants in a few genes, the majority of pustular manifestations remain to be associated with any known genetic aberration [46–51]. The overlap of reported variants between the various subtypes of pustular diseases suggest a shared genetic underpinning. After the discovery of variants in the IL–36 pathway, GPP was considered a monogenic disease. Later, the increased occurrence of variants in more than one disease gene in the same individuals with GPP indicated oligogenic inheritance [50].

**Figure 1:**
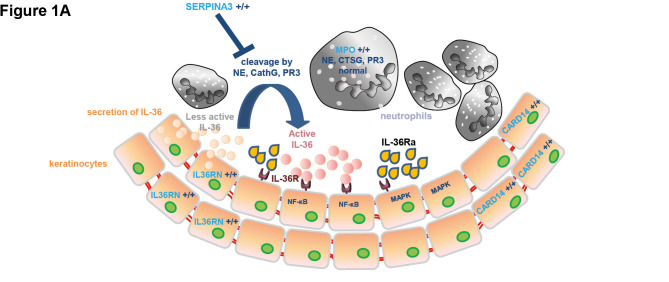

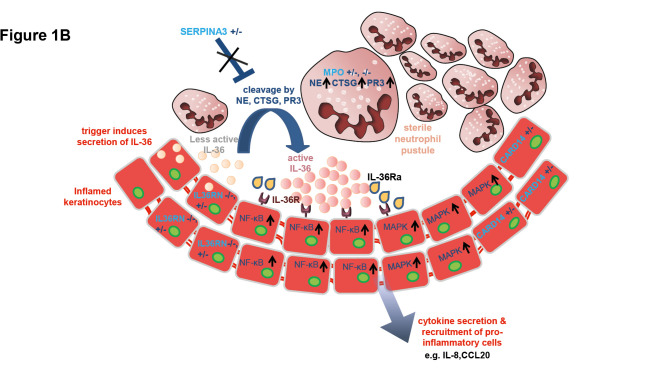
**Model of deficiencies of IL–36Ra, MPO and SERPINA3 in generalized pustular psoriasis.** (A) In physiological state, neutrophils are present in blood and hardly in skin; only small amounts of IL–36 cytokines are produced in keratinocytes and can be cleaved to more active forms by neutrophilic serine proteases (neutrophilic elastase, cathepsin G, proteinase 3). Usually, epidermal SERPINA3 and other serine protease inhibitors inhibit these enzymes. IL–36Ra opposes active IL–36, which is key to maintain homeostasis. *CARD14* is also expressed in keratinocytes. (B) In disease state, neutrophils that are more active migrate to the skin to form sterile epidermal clusters (pustules). Triggers induce the secretion of IL–36. Loss of IL–36Ra or an impaired function of this antagonist leads to increased amounts of IL–36 cytokines causing downstream activation of NF-kB and mitogen-activated protein kinase pathways. These in turn lead to higher levels of IL–8 in keratinocytes. Both mechanisms contribute to increased amounts of active IL–36 and the persistence of epidermal pustules, thereby further increasing pro-inflammatory downstream mediators. Combinations of variants in the two disease genes *IL36RN* and *MPO* are not uncommon, while other combinations have hardly been reported. Triggers lead to changes of CARD14’s localisation in keratinocytes; CARD14 induces activation of NF-kB leading to the transcription of pro-inflammatory genes including IL–8 and CCL20. Proteins translated by these genes recruit further inflammatory cells which in turn produce cytokines that leads to inflammation and further keratinocyte activation. Abbreviations: CARD14: Caspase Recruitment Domain Family, Member 14, CTSG: cathepsin G; IL–36: interleukin–36; IL–36R: interleukin–36 receptor; IL36RN: interleukin–36 receptor antagonist gene; IL–36Ra, interleukin–36 receptor antagonist; IL–8: interleukin–8; MPO: myeloperoxidase; NE: neutrophilic elastase; PR3: proteinase 3; SERPINA3: serine proteinase inhibitor 3. This figure represents a modified and extended Figure 5 published previously by Haskamp et al. [50].

Autosomal recessive *loss-of-function* (LOF) missense variants in ***IL36RN*** were discovered using linkage as well as exome sequencing in both familial and sporadic cases in Tunisian and European populations [46, 47]. *IL36RN* encodes the protein interleukin–36 receptor antagonist (IL–36Ra) which opposes three interleukin–1 family proteins (IL–36α, –β and –γ) expressed in keratinocytes, activating several pro-inflammatory signalling pathways such as NF-kB and mitogen-activated protein kinase pathways (Figure 1) [52, 53]. An over-expression of IL–36α, –β and –γ l in both lesional and non-lesional skin of patients with bi-allelic *IL36RN* mutations lead to higher levels of IL–8 in keratinocytes [46]. Autosomal recessive *IL36RN* variants have been reported in proportions of 20–25 % of patients in studies with predominantly European participants; apart from a younger age of onset in carriers of variants, no genotype-phenotype correlation has been observed until now [14, 54].

17–20 % of ACH cases carry autosomal recessive or heterozygous *IL36RN* variants and about 4 % of AGEP patients heterozygous ones, highlighting the overlap of genetic risk factors [14, 55, 56].

The discovery of pathogenic *IL36RN* variants was a major milestone in the search for effective treatment for psoriasis patients, particularly those with pustular psoriasis. Based on the pathophysiology of the IL–36 pathway and successful animal studies, antibodies against the IL–36 receptor were explored as a potential therapeutic substance [52, 57]. Subsequent clinical trials showed successful treatment of patients with GPP including those without *IL36RN* variants [58, 59]. This example emphasizes the essential potential of genetic research.

A *de novo* variant (c.413A>C/p.(Glu138Ala)) in ***CARD14*** was discovered in a single case of early onset sporadic GPP; *in vitro* studies suggested an upregulation of NF-kB in transfected HEK293 cells and of pro-inflammatory mediators (*CCL20*, *IL8*) in primary keratinocytes (Figure 1) [30]. In a Japanese cohort of GPP patients, c.526G>C/(p.Asp176His) was discovered as a significant risk factor for GPP with concomitant PsV, but neither with GPP nor PsV alone, suggesting that GPP with PsV is genetically different from simple GPP [49]. Significant association of the same variant with GPP was present in Asian populations and shown to affect spontaneous protein oligomerization [60]. To the best of our knowledge, no *CARD14* variants were reported in either ACH or AGEP cases [61].

Starting several decades ago, genetic variants in the gene myeloperoxidase (***MPO***) leading to enzyme deficiency were reported in GPP patients, but usually in single cases, thus making it difficult to consider it as a susceptibility gene. Recent studies discovered bi-allelic variants in *MPO* in cohorts of GPP, ACH and AGEP patients [50, 51]. All variants were LOF variants leading to partial/total enzyme deficiency in the homozygous states. Upon inclusion of heterozygous carriers, association of *MPO* variants leading to total enzyme deficiency with GPP was more significant than of those leading to partial enzyme deficiency [50]. Bi-allelic *MPO* variants are present in ~5 % of GPP patients and seem to play a larger role in GPP than in ACH or AGEP [50, 51]. The occurrence of variants in both genes, *MPO* and *IL36RN*, was higher than expected by chance. The mutational dose of variants in both disease genes correlated inversely with an earlier age of onset.

The protein MPO belongs to the haem peroxidase-cyclooxygenase superfamily. *MPO* is highly expressed in neutrophils and, to a lesser extent, in monocytes. Enhanced activities of neutrophil proteases, thought to activate IL–36 precursors in patients (Figure 1), a reduced phagocytosis of neutrophils by monocytes, a minimised formation of NETs induced by one stimulus and altered apoptotic properties of immune cells have been suggested to contribute to GPP in MPO-deficient patients [50, 51].

The role of two further disease genes in GPP is less clear: Association of more common heterozygous variants in ***AP1S3*,** encoding AP–1 complex subunit σ1C, with pustular psoriasis was reported in Europeans (GPP, ACH and PPP) [62], but never confirmed in independent European individuals and absent in African or Asian individuals. The variants were predicted to destabilize the 3D structure of the AP–1 complex and knockout experiments in cell lines showed a disruption of endosomal translocation of the pattern-recognition receptor, Toll-like receptor 3 [62]. Heterozygous truncating variants in ***SERPINA3*** variant were identified in single GPP patients; functional studies suggest a reduced inhibition of serine proteases leading to increased active pro-inflammatory IL–36 (Figure 1) [63].

## Candidate genes in PPP

PPP is genetically distinct from PsV, as becomes evident from the lack of association with the main PsV susceptibility locus **PSORS1** [64, 65]. Doubtfully, four genes were suggested to be involved in the pathogenesis of PPP. While association of rare variants in ***IL36RN*** with PPP was reported by few groups (in European, mainly European and Chinese individuals, respectively) [14, 55, 66], this could not be confirmed by others (in Chinese, Japanese and European patients, respectively) [65, 67, 68]. So far, association of seven rare missense variants in ***CARD14*** mainly in the coiled-coil and PDZ domains with PPP was discovered, though with opposite effects of variants in the two domains in *in vitro* experiments. Variants in the coiled-coil domain caused an aggregation of insoluble CARD14, leading to a *gain-of-function* phenotype, while variants in the PDZ domain lead to reduced CARD14, resulting in a LOF phenotype. These differential findings suggest that a carefully balanced level of CARD14 activity is necessary for skin immune homeostasis [69]. Two low-frequency missense variants in ***AP1S3*** were reported to be associated with PPP in mainly British individuals [62, 70], while analyses in larger study groups did not confirm this association [54]. Although GPP patients with ***MPO*** variants have been shown to develop concomitant PPP more often than non-carriers [50], follow-up studies in various psoriatic subtypes reported weak evidence for association with PPP not withstanding correction for multiple testing [71]. Findings in *CARD14* and *MPO* still need confirmation in independent studies. Overall, the entirety of disease-relevant variants points towards a minor influence of the so far identified genes and indicate the need for systematic approaches to identify susceptibility factors in PPP.

**In conclusion,** genetic studies in psoriasis lead to the detection of many disease-relevant pathways and mechanisms, which are to some extent also relevant for treatment options. The current knowledge of susceptibility factors explains about a third of heritability in complex subtypes and about a third of cases with generalized pustular psoriasis. While the genetic contribution in complex forms indicates a main role of adaptive immunity e. g. T cell mechanisms, the role of innate immunity is predominant in generalized pustular psoriasis. The far from completely detected genetic underpinnings indicate the need for further genetic research.

In the future, more integrative approaches combining multiple methods and data types e. g. genetic variants from whole exome and whole genome sequences, analyses of chromatin structure and accessibility and tissue-specific expression at the quantitative and qualitative level in disease-relevant tissues might lead to a broader understanding of the genetics of the different psoriatic subtypes. Subsequently, stepping down to a single cell resolution such as single-cell RNA-sequencing of keratinocytes and involved immune cells are possible strategies that might open new windows into the genetic underpinnings of psoriatic diseases.
